# A multimodal imaging approach to the evaluation of post-traumatic epilepsy

**DOI:** 10.1007/s10334-012-0316-9

**Published:** 2012-05-17

**Authors:** Silvia F. Storti, Emanuela Formaggio, Enrica Franchini, Luigi G. Bongiovanni, Roberto Cerini, Antonio Fiaschi, Christoph M. Michel, Paolo Manganotti

**Affiliations:** 1 Department of Neurological, Neuropsychological, Morphological and Movement Sciences, Section of Clinical Neurology, University of Verona, Policlinico G.B. Rossi, P.le L.A. Scuro 10, 37134 Verona, Italy; 2 Department of Neurophysiology, IRCCS San Camillo, Venice, Italy; 3 Department of Pathology and Diagnostics, University of Verona, Verona, Italy; 4 Functional Brain Mapping Laboratory, Department of Fundamental Neurosciences, University Medical School, University of Geneva, Geneva, Switzerland

**Keywords:** Post-traumatic epilepsy, EEG-fMRI, Source localization

## Abstract

**Object:**

Electroencephalography-functional magnetic resonance imaging (EEG-fMRI) coregistration and high-density EEG (hdEEG) can be combined to map noninvasively abnormal brain activation elicited by epileptic processes. By combining noninvasive imaging techniques in a multimodal approach, we sought to investigate pathophysiological mechanisms underlying epileptic activity in seven patients with severe traumatic brain injury.

**Materials and methods:**

Standard EEG and fMRI data were acquired during a single scanning session. The EEG-fMRI data were analyzed using the general linear model and independent component analysis. Source localization of interictal epileptiform discharges (IEDs) was performed using 256-channel hdEEG. Blood oxygenation level dependent (BOLD) localizations were then compared to EEG source reconstruction.

**Results:**

On hdEEG, focal source localization was detected in all seven patients; in six out of seven it was concordant with the expected epileptic activity as defined by EEG data and clinical evaluation; and in four out of seven in whom IEDs were recorded, BOLD signal changes were observed. These activities were partially concordant with the source localization.

**Conclusion:**

Multimodal integration of EEG-fMRI and hdEEG combining two different methods to localize the same epileptic foci appears to be a promising tool to noninvasively map abnormal brain activation in patients with post-traumatic brain injury.

## Introduction

Post-traumatic epilepsy, a common complication of traumatic brain injury (TBI), occurs in 15–20 % of patients with severe brain trauma. The risk of epilepsy is higher during the first 2 years after both mild and severe TBI, and it remains elevated for more than 10 years after the injury. Clinical seizures are often unrelated to the diffuse and severe brain lesions. And because neurosurgical treatment is generally unfeasible, correlations between electroencephalography (EEG) activity, clinical seizures and brain lesions are rarely investigated.

The combined use of EEG and functional magnetic resonance imaging (fMRI) techniques in epilepsy allows for the noninvasive hemodynamic characterization of interictal activity. EEG-fMRI recording can provide information on the pathophysiological processes underlying interictal activity, since the hemodynamic changes are a consequence of the abnormal neural activity generating interictal epileptiform discharges (IEDs). In detail, fMRI measures the hemodynamic response induced by neural activity and can be used to display the active brain regions associated with a process. fMRI does not directly measure neural activity but rather the changes in oxygenation and blood flow. The advantages of fMRI include high spatial resolution (order of mm) and whole brain coverage. Moreover, fMRI combined with standard neurophysiological stimulation techniques can provide important clinical insights into epileptic processes. Conversely, EEG measures with high temporal resolution the neural activity from the scalp generated by brain current sources activity, producing a current dipole moment in each tissue volume [40]. For decades EEG has been used to study and characterize pathophysiologic processes in the brain, but it has a limited ability to localize activity to a deep brain region. Used separately, EEG and fMRI provide low spatial (EEG, order of cm) or temporal (fMRI, order of s) resolution, but when combined they have complementary features. Used simultaneously, EEG and fMRI is a powerful and noninvasive method to investigate the spatiotemporal mechanisms of abnormal brain activity and to precisely define the irritative zone. The relationship between neuronal physiology and the observed blood oxygenation level dependent (BOLD) activity is still unclear. Research results so far suggest a predominantly linear coupling between BOLD and neuronal activity [32, 41].

These technologies were combined by [24] and are widely used in studies of epileptic patients to localize epileptogenic foci [1, 4, 5, 7, 12, 19, 25, 26, 28–31, 33, 34, 42, 46]. The results have been corroborated in several important reviews [37, 45, 47].

The conventional analysis of EEG-fMRI data relies on the general linear model (GLM). The neurologist visually identifies IEDs, and the convolution of these EEG events with a model of the fMRI response, i.e., the hemodynamic response function (HRF), provides the regressor for GLM analysis of fMRI data. The significance in activation is then tested by means of a *t* test which compares the signal intensity of IEDs with that of resting status. In the absence of a paradigm design, a useful alternative is a multivariate approach/analysis such as independent component analysis (ICA). This important technique of data-driven analysis can be applied to solve the problem of blind source separation of signals by dividing the imaging data into several spatial patterns or independent activation maps. ICA can also extract task-related as well as physiologically-relevant non-task-related components and artifactual components. Although ICA decomposition in fMRI is widely used to identify networks, a gold standard selection criteria to separate the ICs related to an internal network from noise-related ICs is still lacking. The existing methods only rank the components, after which the neurologist must scroll each component manually. Nonetheless, ICA decomposition is extremely useful for identifying IED-related activity [39] and the resting state networks (RSNs) of neuronal activity as regions involved in motor function, visual and auditory processing, memory, executive functioning and the default mode network (DMN). In order to identify the IED-related ICs, the design matrix constructed with the GLM can be compared with the components by computing the cross correlation between them.

Another commonly employed technique in the evaluation of epilepsy is electrical source imaging (ESI). ESI can estimate the localization within the brain volume of the electric source(s) generating an IED that can be recorded with scalp electrodes. ESI involves numerous scalp electrodes, high-density EEG (hdEEG), and realistic head models derived from structural MRI. The assumption about the number of electrodes, geometric and anatomical properties, conductive volume and focus characteristics can enhance the precision of the inverse solution [38]. Source localization can be used as part of evaluation of patients with focal epilepsy.

In contrast to standard EEG, hdEEG is a recording method that relies on numerous electrodes to increase spatial sampling. Also, hdEEG can be combined with realistic head models and used in source analysis to determine the generators of IED. To date, few researchers have been able to acquire both signals that could reproducibly demonstrate a correspondence between the EEG-fMRI signal and the electrical sources [56, 58], given that the generators of the EEG and the BOLD signals do not always completely overlap. A recent review of the combined use of EEG-fMRI coregistration and EEG source analysis for event localization in epilepsy reported promising perspectives for these strategies [57].

In this study, we combined noninvasive imaging techniques in a multimodal approach to investigate the path- ophysiological mechanisms of epileptic activity in patients with severe TBI. Here, we describe seven cases of pharmacoresistant post-traumatic epilepsy. Assessment was performed using a multimodal approach with the use of hdEEG and EEG-fMRI technologies. The multimodal approach consists of the source localization applied to 256 EEG-channel data and GLM and ICA analysis of the EEG-fMRI data. Finally, the data were independently analyzed and the results compared by verifying the concordance between EEG source localization and BOLD response to interictal spikes.

## Materials and methods

### Patients

The study sample was seven patients with post-traumatic epilepsy. Clinico-pathological characteristics were roughly similar: complex partial seizures (focal) and seizure frequency (from 3 to 25 seizures per month); 5 out of 7 patients presented with cognitive and behavioral impairment; all showed focal activity, spike and slow-wave paroxysms. Paroxysmal activity predominated in the left frontal regions (5/7 patients), in the right-temporal regions (1/7) and in the left-central regions (1/7).

#### ***Patient No. 1***

 This 43-year-old man sustained severe TBI in a road accident. The first seizures developed 2 years later, with initial clonic movements of the right arm and hand, followed by right leg and orofacial automatisms of the right side of the face, deflection of the face rightward, and early loss of consciousness. MRI revealed left brain cortical and subcortical atrophy with considerable ex vacuo enlargement of the lateral ventricles. Interictal standard scalp EEG over the right hemisphere was inconstant; low voltage (8–9 Hz) EEG over the left hemisphere showed theta waves associated with polymorphic delta waves which were more evident on the fronto-central hemisphere electrodes; specifically, high amplitude (75–120 μV) 2 Hz spike and wave discharges over the left frontal hemisphere electrodes which spread to the right hemisphere.

#### ***Patient No. 2***

 This patient suffered head trauma at age 16 years, with bleeding in the left fronto-polar and frontal regions. Two years later, the first seizure occurred during the night and was described as grand mal seizures with tonic clonic movements of the upper and lower limbs. These were followed by 2–3 episodes of cyclic clinical tonic-clonic seizures per week. Seizure occurrence eventually ceased with additional therapy. Standard EEG showed theta-wave activity in the left frontal regions with phase reversal in F3. Other seizures were characterized by abrupt loss of consciousness, fixed gaze, facial contraction and mild head version to the right.

#### ***Patient No. 3***

 At the age of 14 years, this patient suffered an accidental fall resulting in extradural hematoma and right hemiparesis which resolved (lasting 3 days). Two apparently generalized tonic-clonic seizures occurred during sleep at age 38 years, in addition to sporadic left lower limb clonic seizures during drowsiness. The patient presented no motor deficit, sensory, cognitive or behavioral impairment. Standard EEG showed slow left fronto-temporal foci (drug-resistant) associated with paroxysmal isolated elements in F7. The patient showed continuous theta activity, sometimes polymorphic delta sequences, of medium voltage on the left frontal electrodes. At the same site, isolated slow peaks were detected mainly in Fp1/F7. Rare and isolated polyspikes associated with left frontal slow waves on derivations were recorded only in stage II NREM sleep. These elements showed phase reversal in F7, without a tendency to spread, and disappeared upon awakening.

#### ***Patient No. 4***

 This patient sustained a fracture of the right cranial vault in a motorcycle accident 12 years earlier. After the accident, he fell into coma for 22 days. The front vault was removed and a prosthesis inserted. MRI findings were principally bilateral frontal lesions. He presented right-sided blindness resulting from trauma-induced lesion of the optic nerve and residual orbitofrontal asymmetry of the facial bones. Interictal standard EEG showed theta polymorphic sequences during drowsiness associated with isolated large-voltage paroxysms on the left fronto-temporal electrodes with phase reversal in F7. These elements did not tend to spread contralaterally. His history included brief attacks typically occurring in clusters and often at night, characterized by oscillatory trunk movements, arm flexion, synchronous bi-manual automatisms, and proximal isolated movements of the lower limbs associated with loss of consciousness.

#### ***Patient No. 5***

 This 44-year-old man suffered a head injury in a workplace accident at the age of 31 years, associated with an acute left fronto-temporo-parietal subdural hematoma, multiple brain injuries and cerebral edema resulting in right hemiplegia. The patient also presented with motor aphasia. Simple focal seizures first occurring 9 months after the accident were characterized by initial deviation of the mouth and eyes to the right, followed by clonic movements of the upper and then the lower right limb, lasting less than 1 min, on an average of 1–2 times a year. MRI showed bilateral lesions in the frontal regions and in the left temporo-parietal regions. EEG recordings were characterized by slow theta, sometimes delta, polymorphic activity intermixed with superimposed sharp waves showing phase reversal to C3.

#### ***Patient No. 6***

 At age 19 years, this patient sustained the first and the most severe head trauma in a road accident, resulting in a left fronto-orbital lesion. Reconstruction of the front and placement of implants were performed. Four years after the accident, the patient experienced two episodes of morpheic seizures. One year later, he sustained another head injury, followed by focal and secondarily generalized seizures (starting with prolonged vocalization and quickly followed by a tonic and then a clonic phase). The last seizure occurred 4 months before the present fMRI study. MRI showed a large left frontal and an orbitofrontal poroencephalic lesion associated with significant gliosis. EEG recordings showed persistent epileptogenic foci, with theta activities intermixed with superimposed spikes and waves in the left frontotemporal regions, with maximum appearance at the F7 electrode.

#### ***Patient No. 7***

 This patient presented bilateral lesions after sustaining a work-related accident (right temporal lesion and left temporal lobe lesion). His clinical picture was characterized by temporal seizures with temporo-parietal aura characterized by auditory hallucinations (“like hearing the sea inside a seashell”) followed by epigastric sensations, nausea and loss of consciousness. He sometimes experienced complex hallucinations (“like a slide series about my past life”) associated with oral automatisms, head version to the right, fixed gaze and frothing at the mouth. The patient showed theta-delta activity with sharp, large-voltage morphology in the fronto-temporal region and phase reversal in T4.

### Data acquisition

#### HdEEG

HdEEG recording was performed using 256 channels (Electrical Geodesic, Inc., Eugene, OR, USA). The net was adjusted so that Fpz, Cz, Oz and the pre-auricular points were correctly placed according to the international 10/20 system. Due to the geodesic tension structure of the net all electrodes were evenly distributed on the scalp at approximately the same location across subjects. The data were recorded against a vertex electrode reference (Cz) at a sampling rate of 200 or 250 Hz and filtered off-line with a band-pass filter (1–30 Hz).

#### EEG-fMRI data

During MRI scanning, EEG data were acquired using an MR-compatible EEG amplifier (SD MRI 32, Micromed, Treviso, Italy) and a cap with 32 Ag/AgCl electrodes positioned according to a 10/20 system. To remove pulse and movement artifacts during scanning, two of the electrodes were used to record the electrocardiogram (ECG) and electromyogram (EMG). The EMG electrode was placed on the right abductor pollicis brevis muscle. The reference was placed anterior to Fz, and the ground posterior to Fz, as previously described elsewhere [15, 51] for the same system. EEG data were acquired at a sampling rate of 1,024 Hz using the SystemPlus software package (Micromed, Italy). To avoid saturation, the EEG amplifier had a resolution of 22 bits with a range of ±25.6 mV. An anti-aliasing hardware band-pass filter was applied with a bandwidth of 0.15–269.5 Hz.

Functional images were acquired on a 1.5 T MR scanner (Symphony, Siemens, Erlangen, Germany) equipped with echo-planar imaging (EPI) capability and a standard transient/receive (TR) head coil. fMRI data were acquired with a *T*2^*^ weighted EPI sequence (36 slices, TR = 3,700 ms, TE = 50 ms, 64 × 64 matrix, FOV = 256 × 256, slice thickness 3 mm; voxel size = 3 × 3 × 3 mm, axial slice orientation). At the start of each fMRI acquisition, the scanner gave a trigger signal that was recorded by the EEG system and used as a volume marker. A T1-weighted anatomical scan (192 slices, TR = 1,990 ms, TE = 3 ms; scanning matrix 512 × 512, FOV = 256 × 256; slice thickness 1 mm; sagittal slice orientation) was also acquired for each patient.

The patients were positioned supine on a bed inside the scanner bore, with elbows flexed at 120° and hands pronated in a relaxed position; the head was stabilized on both sides with adjustable padded restraints. During fMRI acquisition, the patients were instructed to keep their eyes closed and remain as still as possible throughout the procedure. A total of 200 volumes were acquired for patient No. 1, and 140 volumes for the other 6 patients; the duration of one volume was 3,700 ms.

### Data analysis

#### Source localization

HdEEG data were analyzed using Cartool software (http://sites.google.com/site/cartoolcommunity/). The T1 anatomical images acquired before each EEG-fMRI session were used to create a realistic model of the brain for source localization. The original MRI is anisotropic. The images were converted in isotropic using Brain Voyager (Brain Innovation, Maastricht, The Netherlands) with a voxel size of 1 × 1 × 1 mm^3^. In each patient, the hdEEG and MRI data and the solution space were restricted to the gray matter. The solution space for the distributed source model contains from a min of 3,010 to a max of 3,032 points uniformly distributed over the gray matter of the brain mapped onto the spherical head model with anatomical constraints (SMAC) space [50]. The SMAC head model method has been successfully employed in several previous clinical and experimental studies [9, 22, 38, 49, 58] and it produces localization precisions comparable with realistic boundary element models [23]. The EEG electrode positions were matched to the MR scalp surface using transformation operations such as rotation and translation according to common landmarks during both EEG and MRI acquisition. The peak of the spike was used as a trigger for averaging in epochs of ±500 ms. The number of spikes ranged from 35 to 42. Two time frames were chosen to characterize spike topography: the first, from the beginning of the spike to the time point corresponding to 50 % of the rising phase as an epoch characterizing a source of the possible spike generator [27]; the second, an epoch at the peak of the spike indicating propagation. Special attention was paid to marking the same type of spikes as those obtained during the recordings inside the scanner. A standardized source imaging procedure, low resolution brain electromagnetic tomography (LORETA) [43] constrained to the individual matter, was applied to the averaged spikes.

#### GLM on EEG-fMRI data

##### EEG pre-processing

The data were processed using Matlab 7 (MathWorks, Natick, MA, USA) and EEGLAB 4.51 (http://sccn.ucsd.edu/eeglab/) [14]. The EEG artifact induced by the magnetic field gradient was digitally removed off-line using an adaptive filter [3], while the EEG artifact associated with pulsatile blood flow, ballistocardiogram, was removed using an averaging procedure [2], both of which were implemented with SystemPlus software (Micromed). Reference-free recordings were then obtained by calculating the local average reference using EEGLAB. A notch filter (50 Hz) was also applied to all channels. EEG recordings were band-pass filtered from 1 to 30 Hz using a FIR filter because all seven patients had a routine EEG recording showing focal IEDs at frequencies above 1 Hz. The signal was then baseline corrected and detrended; epochs with artifacts were visually identified and excluded from the analysis.

##### fMRI pre-processing

Before statistical analysis, the functional data were pre-processed using Brain Voyager software. The MR images were realigned to reduce the effect of head motion (three-dimensional motion correction with sinc interpolation). To correct for the different acquisition times, slice scan time correction was used in an ascending, interleaved scanning order with linear interpolation over time. The data were then pre-processed with linear trend removal to remove all drifts, and with a temporal high-pass filter (three cycles in time course) to reduce the effect of breathing and physiological noises. The anatomical and functional data were kept in the subject’s native space. The optional temporal smoothing filter was not used during analysis, while spatial Gaussian smoothing was used with Gaussian kernel 6 mm full width at half maximum (FWHM).

##### GLM analysis

The analysis of EEG-fMRI data was based on the visual identification of the IEDs on scalp EEG. The convolution of these EEG events, represented as stick functions, with the HRF provided the regressor for GLM analysis of fMRI data. Activated voxels were identified with a single-subject GLM approach for the time series data [17]. Confounds were included to account for motion-related effects. Temporal autocorrelations were accounted for by fitting an autoregressive (AR) model. Noise was modeled as an AR process estimated from the residuals by using Yule–Walker equations. The partial autocorrelation plots were examined to identify the AR order. A statistical significance was found for lag 1, indicating that the AR model was appropriate for all patients, except for patient No. 3. The confidence interval for statistical significance was of 95 %. Brain activation was detected by comparing the signal intensity of active images (IED activity) with that of resting images based on the changes in local BOLD signals and on the voxel-wise Student’s *t* test (with FDR correction according to the number of voxels related to the brain volume). *Z*-score maps representing brain activation were then generated from *t*-maps. The results were displayed as parametric statistical maps, where the pixel *Z* value is expressed on a colorimetric scale.

#### PICA on EEG-fMRI data

##### fMRI pre-processing

The functional data were pre-processed using Multivariate Exploratory Linear Decomposition into Independent Components (MELODIC) version 3.09, a part of the FSL toolbox (http://www.fmrib.ox.ac.uk/fsl). The images were smoothed with a Gaussian kernel 6 mm (FWHM) and motion-corrected. To correct for the different acquisition times, an interleaved slice timing correction was used. The data were then pre-processed with high-pass temporal filtering (cut-off of 100 s) and with the removal of non-brain structures from the echo planar imaging volumes (Brain Extraction Tool [BET] http://www.fmrib.ox.ac.uk/fsl).

##### PICA analysis

The pre-processed data underwent probabilistic independent component analysis (PICA) decomposition using MELODIC. The maps were thresholded at a posterior probability threshold of *p* > 0.5 [6]. A threshold of *z* scores was used to visualize the IC maps. From a variable number of ICs obtained by PICA decomposition (range 13–36 components), the components were then visually selected by an expert neurologist. In order to identify which components were related to a patient’s IED activity, the design matrix constructed in the GLM analysis was fitted to the time course of each component. Components significantly related to IED activity were identified with an *F* test (*p* < 0.05, corrected for the number of components) [39]. Statistically significant *F* values identify ICs time courses showing significant signal changes time-locked to the IED activity.

## Results

### Comparison of ESI with EEG-fMRI results

Clinical information and results for each patient are summarized in Table 1. A homogeneous population was studied to explore methodological issues. This group of post-traumatic epilepsy patients was consistent in its number with some clinico-pathological correlations.

**Table 1:** Clinical profiles

Pt	Age	Sex	Trauma	1st seizure	Drugs	EEG	p.r.	Lesion	Imaging	IED localization	
										ESI	EEG-fMRI
1	43	M	Car accident	2 years	VPA 550 mg/die, ZNS 325 mg/die, LEV 4,000 mg/die, OXC 2,100 mg/die, PB 100 mg/die	Low voltage on the fronto-central hemisphere electrodes	F3	Left brain damage	Left cerebral atrophy, poroencephalic lesions and gliosis	Left frontal and central regions	Activation of left central region, SMA and deactivation of the frontal cortex
2	32	M	Car accident	2 years	VPA 2,500 mg/die, OXC 1,800 mg/die	Slow waves activity in left frontal regions	F3	Bilateral frontal brain lesions	Left frontopolar poroencephalic cysts with atrophy and gliosis	Frontal lobe	No BOLD activation
3	46	M	Accidental fall	24 years	LEV 2,000,mg/die	Slow left fronto-temporal foci	F7	Left frontal extradural hematoma	Left frontal poroencephalic cyst	Left front-mesial lobe (drowsiness)	No BOLD activation
4	27	M	Motorcycle accident	1 year	CBZ 1,400 mg/die, ZNS 475 mg/die, PB 125 mg/die	Theta polymorphic sequences on the left fronto-temporal electrodes	F7	Fracture of the right frontal cranium	Alteration of signal intensity on the left but mainly right frontal lobes	Right front lobe	Frontal lobe
5	44	M	Work accident	9 months	OXC 1,800 mg/die, LEV 500 mg/die, PB 100 mg/die	Slow theta and delta sequences with isolated components with sharp morphology	C3	Left fronto-temporo-parietal acute subdural hematoma, multiple brain lesions and cerebral edema	Left temporo-occipital poroencephalic cyst, hypodense areas in bilateral frontal and left temporo-parietal regions	Left central posterior electrodes	No BOLD activation
6	57	M	Car accident	4 years	PHT 400 mg/die, Clonazepam 2 mg/die	Theta slow sequences on left fronto-temporal electrodes	F7	Fracture of the left frontal cranium	Large poroencephalic lesion in the left frontal and fronto-orbital areas with gliosis	Left frontal-central-temporal lobe	Activation in frontal area and deactivation in left frontal area
7	57	M	Accidental fall	1 year	OXC 1,800 mg/die	Theta–delta activity on the fronto-temporal areas	T4	Right skull fracture, right acute subdural hematoma and bitemporal lesions	Pore cavity of the brain with atrophy and gliosis perifocal and expansion bilaterally of the ex-vacuo temporal ventriculi	Right temporal lobe	Right temporal lobe

*Pt* patient, *1st seizure* first seizure from injury, *p.r*. phase reversal of EEG

On hdEEG, focal source localization was detected in all patients. In six out of seven patients, it was concordant with the expected epileptic activity as defined by EEG data and clinical evaluation. BOLD signal changes were observed in four out of seven patients in whom IEDs were recorded (patients Nos. 1, 4, 6 and 7). These activities were concordant with the source localization in patients Nos. 1, 6 and 7 and only partially concordant in patient No. 4. In some patients, the IEDs inside the magnet were not very frequent and of limited duration, which subsequently created difficulties in the off-line analysis. ICA identified IED-related activity in three out of seven patients (patients Nos. 1, 4, and 6). DMN was not always detected (i.e., patients Nos. 1 and 5).

### Single-subjects results

#### ***Patient No. 1***

**Fig. 1:**
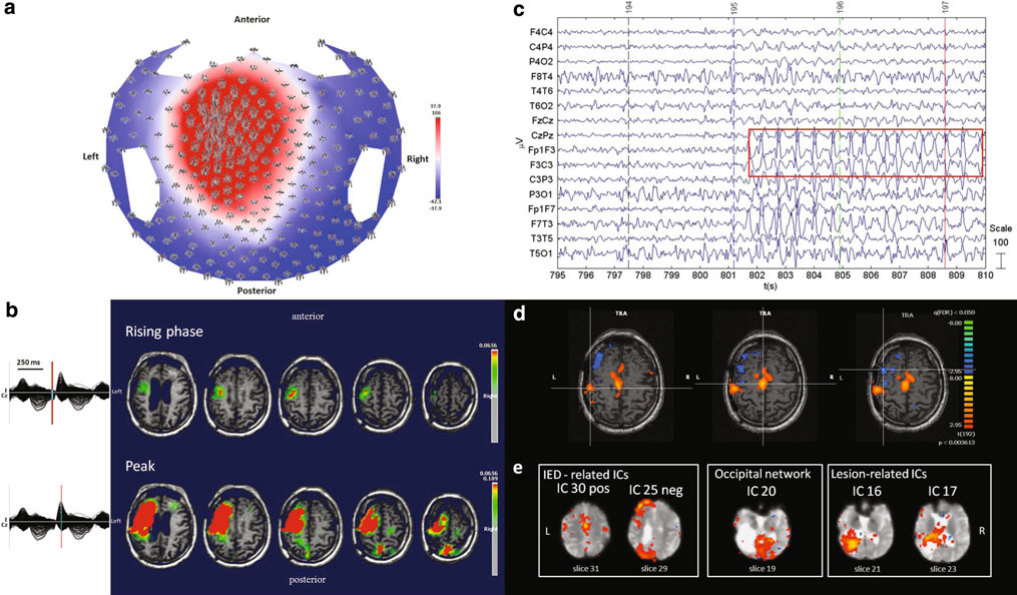
Patient No. 1. **a** HdEEG signal visualized according to the projected locations of the scalp electrodes (spike average). **b** HdEEG source analysis results. On the *left*, 256-channel EEG traces with a duration of 1 s (spike average). The global field power is used for the onset (*red line*). On the *right*, EEG source imaging in the rising phase of the peak and at the peak. The maximum of the estimated source of the average interictal spike is indicated in *red*. **c** EEG acquired during fMRI scanning after preprocessing. **d** EEG-fMRI results from the GLM analysis. Corrected *p* value (FDR, *p* < 0.05) is visualized, and the *color bar* shows the *Z* score scale. **e** EEG-fMRI results from the ICA analysis. The maps were thresholded at a posterior probability threshold of *p* > 0.5

 HdEEG activity showed sequences of spike and wave complexes over the left fronto-central regions lasting more than 10 s (Fig. 1a). The source localization showed a defined localization over the rolandic areas compatible with focal EEG activity (Fig. 1b). EEG activity inside and outside the magnet showed a high amplitude over the left fronto-polar electrodes, with a phase reversal over the frontal and central electrodes (Fig. 1c). The GLM analysis showed prominent BOLD activation over the left central cortical areas (rolandic and sylvian) adjacent to the brain damage and anterior to the ventricular enlargement and over the supplementary motor areas (SMA) during the spikes and wave discharges as compared to the resting state. A deactivation in the left frontal and parietal regions, not observed in the source analysis, was also obtained, FDR (*q* < 0.05) (Fig. 1d). The PICA decomposition estimated 36 components, five of which were related to internal processes. Components nos. 30 and 25 were significantly IED-related ICs: the first was positively correlated with the GLM design matrix and the second negatively (*F* test, *p* < 0.05, corrected for the number of components); component no. 20 represented the occipital network; components nos. 16 and 17 the lesion-related ICs. The DMN was not detected (Fig. 1e).

#### ***Patient No. 2***

**Fig. 2:**
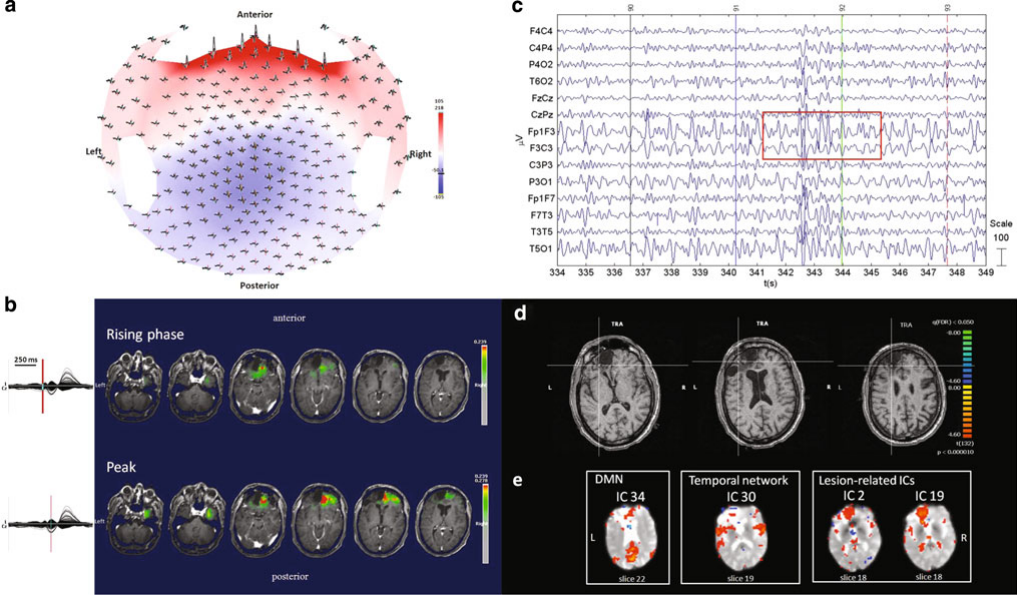
Patient No. 2. **a** HdEEG signal visualized according to the projected locations of the scalp electrodes (spike average). **b** HdEEG source analysis results. On the *left*, 256-channel EEG traces with a duration of 1 s (spike average). The global field power is used for the onset (*red line*). On the *right*, EEG source imaging in the rising phase of the peak and at the peak. The maximum of the estimated source of the average interictal spike is indicated in *red*. **c** EEG acquired during fMRI scanning after preprocessing. **d** EEG-fMRI results from the GLM analysis. Corrected *p* value (FDR, *p* < 0.05) is visualized, and the *color bar* shows the *Z* score scale. **e** EEG-fMRI results from the ICA analysis. The maps were thresholded at a posterior probability threshold of *p* > 0.5

 HdEEG showed slow-wave activity in the perilesional frontal and prefrontal regions, consistent with the head trauma (Fig. 2a). The source localization showed a defined localization over the frontal region (Fig. 2b). The EEG acquired inside the scanner showed predominant left frontal activity (Fig. 2c). The EEG-fMRI activation maps showed no significant activation, probably due to the short duration of sequences characterized by IED activity, FDR (*q* < 0.05) (Fig. 2d). The PICA decomposition estimated 35 components, four of which were related to internal processes: the DMN (component no. 34); the temporal network, a network primarily including the bilateral superior temporal cortex, corresponding to the auditory-phonological system (component no. 30); and two components related to the lesion area (components nos. 2 and 19). No IED-related IC was identified (Fig. 2e).

#### ***Patient No. 3***

**Fig. 3:**
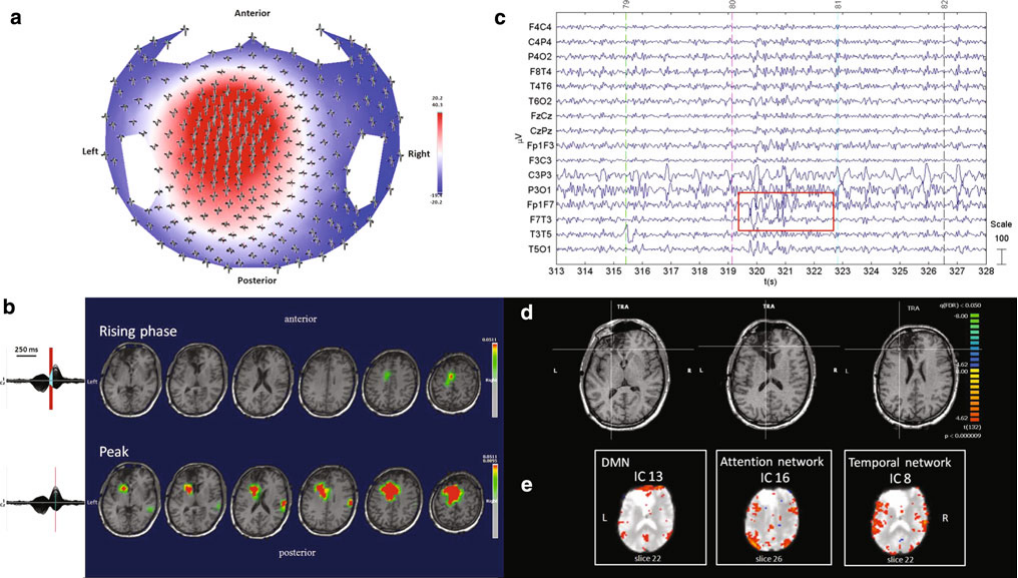
Patient No. 3. **a** HdEEG signal visualized according to the projected locations of the scalp electrodes (spike average). **b** HdEEG source analysis results. On the *left*, 256-channel EEG traces with a duration of 1 s (spike average). The global field power is used for the onset (*red line*). On the *right*, EEG source imaging in the rising phase of the peak and at the peak. The maximum of the estimated source of the average interictal spike is indicated in *red*. **c** EEG acquired during fMRI scanning after preprocessing. **d** EEG-fMRI results from the GLM analysis. Corrected *p* value (FDR, *p* < 0.05) is visualized, and the *color bar* shows the *Z* score scale. **e** EEG-fMRI results from the ICA analysis. The maps were thresholded at a posterior probability threshold of *p* > 0.5

 HdEEG data acquisition lasted 40 min; during drowsiness the patient showed activity with a maximal amplitude over the left central and frontal regions (Fig. 3a). The source localization showed left frontal mesial activity (Fig. 3b). The EEG acquired inside the magnet showed predominant activity over the left frontal electrodes, with a phase reversal in F7 (Fig. 3c). No BOLD activity was detected with GLM analysis, FDR (*q* < 0.05) (Fig. 3d). The PICA decomposition estimated 18 components, three of which were related to internal processes: the DMN (component no. 13); the attention network (component no. 16); and the temporal network (component no. 8). No IED-related IC was identified (Fig. 3e).

#### ***Patient No. 4***

**Fig. 4:**
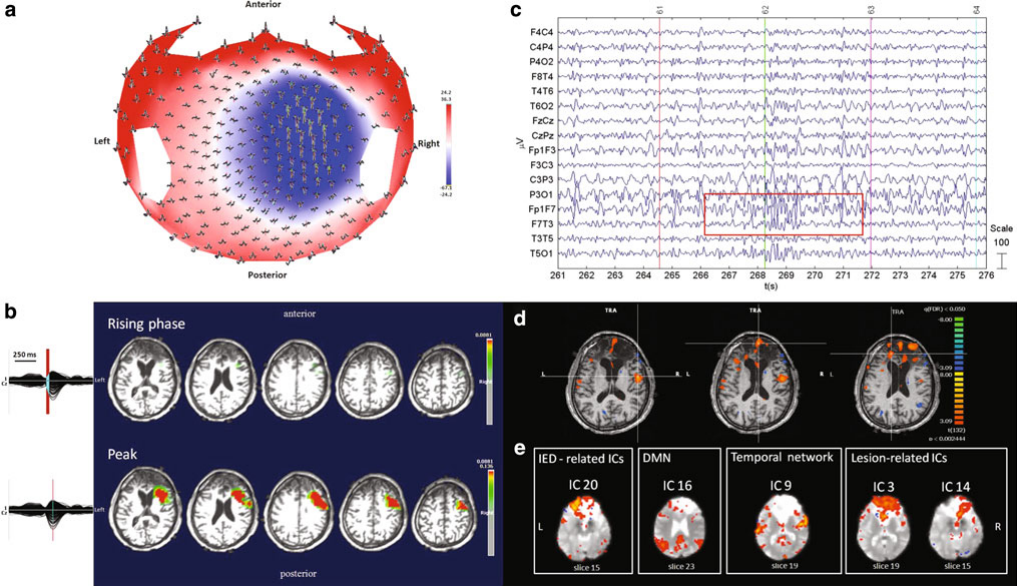
Patient No. 4. **a** HdEEG signal visualized according to the projected locations of the scalp electrodes (spike average). **b** HdEEG source analysis results. On the *left*, 256-channel EEG traces with a duration of 1 s (spike average). The global field power is used for the onset (*red line*). On the *right*, EEG source imaging in the rising phase of the peak and at the peak. The maximum of the estimated source of the average interictal spike is indicated in *red*. **c** EEG acquired during fMRI scanning after preprocessing. **d** EEG-fMRI results from the GLM analysis. Corrected *p* value (FDR, *p* < 0.05) is visualized, and the *color bar* shows the *Z* score scale. **e** EEG-fMRI results from the ICA analysis. The maps were thresholded at a posterior probability threshold of *p* > 0.5

 HdEEG showed high activity over the right frontal electrodes, as confirmed by the source analysis (Fig. 4a, b). EEG inside the MR scanner showed high activity on the frontal electrodes (Fig. 4c). The patient underwent two continuous EEG-fMRI sessions. During the first fMRI scanning, a spontaneous critical episode lasting about 41 s was recorded; it was characterized by arm lifting and flexion, synchronous bi-manual automatisms, and ended with extension and repositioning of the limbs on the chest. Isolated synchronous movements of the lower limbs occurred towards the middle of the critical episode. The first delta rhythmic sequences and later muscle and motion artifacts suddenly appeared in the right frontal lobe. During attenuation of the motor phenomena, delta sequences were recorded in the left frontal regions. The second fMRI scan was recorded during interical events and used for the analysis (Fig. 4c). BOLD activity was perilesional and localized over the frontal area, FDR (*q* < 0.05) (Fig. 4d). The PICA decomposition estimated 22 components, five of which were selected: one related to IED activity (component no. 20); one related to the DMN (component no. 16); one related to the temporal network (component no. 9); and two related to the area of the lesion (components nos. 3 and 14) (Fig. 4e).

#### ***Patient No. 5***

**Fig. 5:**
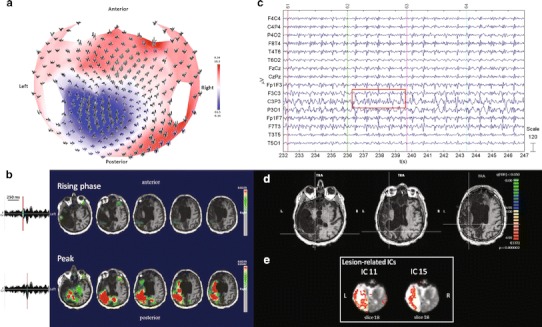
Patient No. 5. **a** HdEEG signal visualized according to the projected locations of the scalp electrodes (spike average). **b** HdEEG source analysis results. On the *left*, 256-channel EEG traces with a duration of 1 s (spike average). The global field power is used for the onset (*red line*). On the *right*, EEG source imaging in the rising phase of the peak and at the peak. The maximum of the estimated source of the average interictal spike is indicated in *red*. **c** EEG acquired during fMRI scanning after preprocessing. **d** EEG-fMRI results from the GLM analysis. Corrected *p* value (FDR, *p* < 0.05) is visualized, and the *color bar* shows the *Z* score scale. **e** EEG-fMRI results from the ICA analysis. The maps were thresholded at a posterior probability threshold of *p* > 0.5

 HdEEG showed high activity over the left central posterior electrodes (Fig. 5a). The source was localized over the left residual rolandic area (Fig. 5b). EEG acquired inside the scanner showed slow activity in the left motor area, with phase reversal in C3 (Fig. 5c). No BOLD activity was detected with GLM analysis, FDR (*q* < 0.05) (Fig. 5d). The PICA decomposition estimated 21 components, two of which related to the area of the lesion (components nos. 11 and 15) were selected. No components related to DMN or IED activity were identified (Fig. 5e).

#### ***Patient No. 6***

**Fig. 6:**
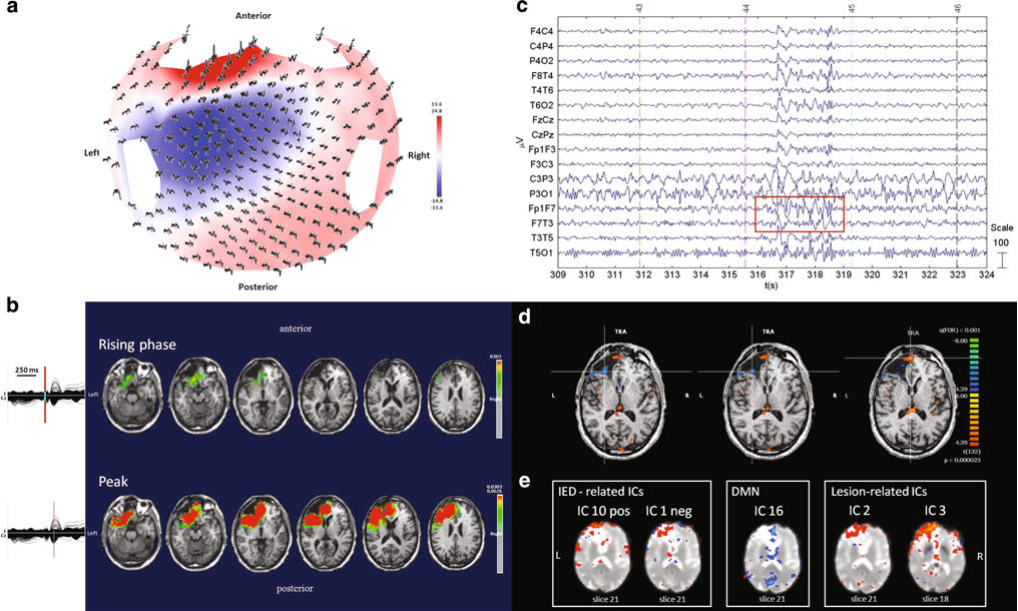
Patient No. 6. **a** HdEEG signal visualized according to the projected locations of the scalp electrodes (spike average). **b** HdEEG source analysis results. On the *left*, 256-channel EEG traces with a duration of 1 s (spike average). The global field power is used for the onset (*red line*). On the *right*, EEG source imaging in the rising phase of the peak and at the peak. The maximum of the estimated source of the average interictal spike is indicated in *red*. **c** EEG acquired during fMRI scanning after preprocessing. **d** EEG-fMRI results from the GLM analysis. Corrected *p* value (FDR, *p* < 0.001) is visualized, and the *color bar* shows the *Z* score scale. **e** EEG-fMRI results from the ICA analysis. The maps were thresholded at a posterior probability threshold of *p* > 0.5

 HdEEG was recorded in the waking state and at initial drowsiness. HdEEG showed high activity over the left fronto-central-temporal area (Fig. 6a). The source analysis showed two focal sources near the lesion: one frontal and more anterior and the other more toward the left side (Fig. 6b). EEG inside the magnet showed a slow sequence on the left fronto-temporal electrodes, particularly over F7 (Fig. 6c). EEG-fMRI showed two main changes: the first was characterized by more anterior activation in the frontal area and the second by more lateralized deactivation in the left frontal area, FDR (*q* < 0.001) (Fig. 6d). The PICA decomposition estimated 19 components, five of which were selected: components nos. 1 and 10 were significantly IED-related ICs; the first was negatively and the second positively correlated with the GLM design matrix (*F* test, *p* < 0.05, corrected for the number of components); component no. 16 was the DMN; components nos. 2 and 3 were lesion-related ICs (Fig. 6e).

#### ***Patient No. 7***

**Fig. 7:**
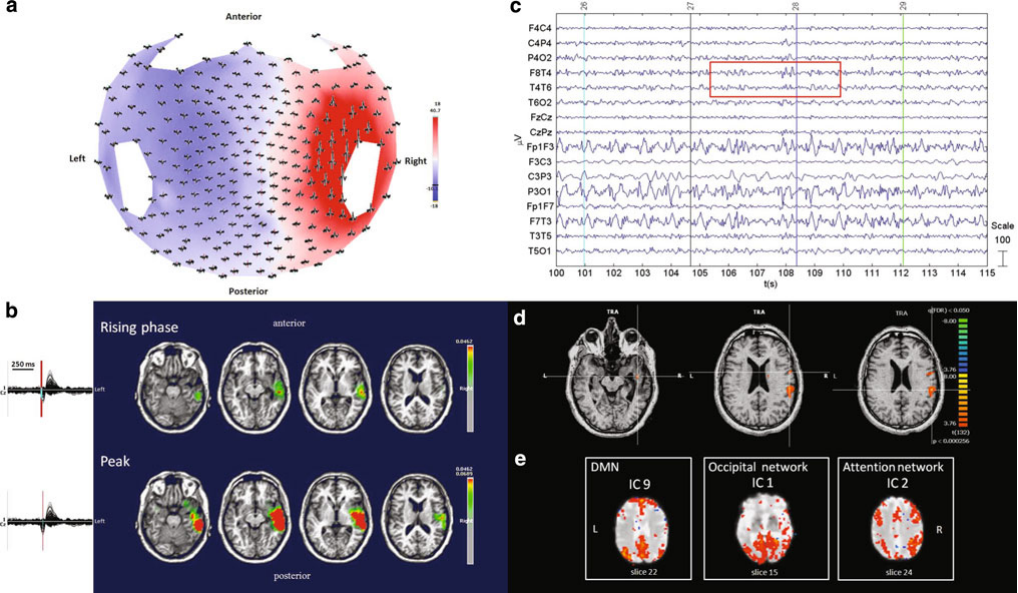
Patient No. 7. **a** HdEEG signal visualized according to the projected locations of the scalp electrodes (spike average). **b** HdEEG source analysis results. On the *left*, 256-channel EEG traces with a duration of 1 s (spike average). The global field power is used for the onset (*red line*). On the *right*, EEG source imaging in the rising phase of the peak and at the peak. The maximum of the estimated source of the average interictal spike is indicated in *red*. **c** EEG acquired during fMRI scanning after preprocessing. **d** EEG-fMRI results from the GLM analysis. Corrected *p* value (FDR, *p* < 0.05) is visualized, and the *color bar* shows the *Z* score scale. **e** EEG-fMRI results from the ICA analysis. The maps were thresholded at a posterior probability threshold of *p* > 0.5

 HdEEG showed maximal amplitude over the right temporal electrodes (Fig. 7a). The source analysis showed a focal source localized in the right temporal lobe (Fig. 7b). The EEG acquired inside the magnet showed predominant activity over the right temporal regions (T4) (Fig. 7c). According to the GLM analysis, the BOLD activity showed an activation in the right temporal lobe and other sparse activations, FDR (*q* < 0.05) (Fig. 7d). The PICA decomposition estimated 13 components, three of which were selected: the DMN (component no. 9); the occipital network (component no. 1); and the attention network (component no. 2). No IED-related IC was identified (Fig. 7e).

## Discussion

Here, we report on a multimodal approach to the assessment of post-traumatic epilepsy, a complicated pharmacoresistant form of epilepsy. Clinical semeiology, BOLD activation and source localization were compared. Seizure semeiology indicated the onset of seizures; MRI showed the diffuse damage that, although a substrate of post-lesional epilepsy, is not specific enough to localize epileptogenic areas close to or far from injured brain tissue. The correlation between brain metabolic activity and scalp electrical spikes has been reported in previous studies on different types of epilepsy [57].

The novelty of this study is the use of a multimodal approach in which hdEEG and EEG-fMRI data on pathophysiological mechanisms underlying post-traumatic epilepsy are compared. The utility of a multimodal imaging approach to localize seizures was investigated by studying the relationship between source localization at different time points and BOLD activity. The ESI results showed one or more sources in all seven patients; and in four out of seven cases, the GLM results showed BOLD activation. EEG-fMRI studies demonstrated that IED-related BOLD signal changes can be observed at the same site and remote from the putative seizure onset zone in patients with cortical lesions. While multiple BOLD activations may be interpreted as a measure of network activation in relation to paroxysmal activity, source localization is able to define a focal activity.

Combined recordings as in EEG-fMRI have the dual advantage offered by the high temporal resolution of EEG and the spatial resolution obtained with neuroradiological exams. EEG-fMRI coregistration is widely used to study the sources of epileptic spikes recorded on the scalp in patients with no visible lesion on MRI [8, 13, 16, 20, 44]. Nevertheless, very few data exist on EEG-fMRI coregistration in post-traumatic epilepsy. Baudewig et al. [5] the first to study a patient with generalized epilepsy after head injury, reported BOLD responses compatible with the clinical picture. Rodionov et al. compared the findings of ICA and EEG-based GLM analyses of fMRI data from eight patients with focal epilepsy. In one patient with post-traumatic epilepsy, a single matching independent component (IC) was found to correspond excellently with the GLM result [46]. More recently, Cosottini et al. studied IED-related BOLD response in a patient with post-traumatic focal epilepsy at repeated EEG-fMRI exams. They found that the BOLD response may be focal or multifocal, depending on the spatial distribution of interictal EEG activity, and that the selection of IED within a well-defined focal EEG field, against bilateral diffuse events, can be helpful in the localization of irritative zones and in improving the reproducibility of results [12].

There are limitations to the technique, however. First of all, there are technical problems associated with the interference between the EEG and MR instruments. During EEG-fMRI acquired simultaneously, EEG is obscured by artifacts induced by both the static and the time-variant fields of the MR scanner. Simultaneous acquisition introduces common artifact on MR images and on EEG. The MR artifacts on images are caused by electrodes, wires and the EEG system inside the magnet room near the scanner. Differently, the EEG artifacts are caused by movement of the leads themselves within the static filed of the magnet that induces an electromotive force in a wire loop (ballistocardiogram artifact). In addition, the radio-frequency pulses and the gradient switching, required for MRI, may induce voltages that obscure the EEG signal (gradient artifact). These common artifacts on MR images and on EEG can be filtered off-line [18].

Other limitations are related to the epilepsy. First, because it is impossible to predict when epileptic discharges will occur, few IEDs will usually appear during the few minutes of fMRI recording; this hampers the possibility to detect BOLD signal changes [48], e.g., in patients Nos. 2, 3 and 5. Second, because of the volume conductor, deep epileptic discharge cannot be captured by scalp EEG [46]. Nonetheless, these limitations can be overcome by applying multimodal and comparative analysis approaches.

In epilepsy evaluation, the most common method to analyze EEG-fMRI data is with the GLM, wherein an a priori hypothesis is made based on the shape of the HRF. With the GLM approach to the evaluation of epileptic patients in resting state, a significant concordance can be observed between focal EEG interictal slow-wave discharges and focal BOLD activation on fMRI.

Different approaches to identifying patterns of coherent activity are used in the analysis of RSNs (for a review see [10]). A method for hypothesis-driven analysis is based on correlating the time course in a certain seed region with the time courses of all other brain voxels. Seed voxel definition, however, is typically based on a priori knowledge of functional localization. Moreover, data-driven analyses have attracted increasing attention: ICA [36]; hierarchical clustering [11]; and Laplacian clustering [52], none of which require a specific prior model definition. In particular, ICA can extract IED-related BOLD components without constraints on the HRF. With ICA, fMRI can be analyzed in the absence of spatial bounds or a priori knowledge about the time course of activation of the different components, whether a component is activated by specific psychophysiological systems or is related to machine noise or other artifacts [36].

The ICA method was first applied to patients with epilepsy by [46]. The components were selected if they showed a spatial overlap with positive BOLD signal changes in the GLM analysis and a correlation of the components time course with the model used in the GLM analysis. More recently, a new method to compare GLM and ICA results was introduced by [39]. In this study, spatial ICA was used to extract ICs from the fMRI data, and a deconvolution method identified component time courses significantly related to the generalized EEG discharges but without imposing constraints on the HRF. In our study, the BOLD data were analyzed by both GLM and ICA. GLM detected significant modifications in four out of seven patients; whereas ICA identified IED-related activity in three out of seven patients, and the time course of the component significantly correlated with the design matrix created by the GLM in patients Nos. 1 and 6 (*F* test, *p* < 0.05 corrected for the number of components). Compared to the GLM, ICA has the added advantage of detecting resting-state networks such as the DMN, the attention network, the posterior network, and the temporal network. The DMN is relevant for two reasons: first, the regions involved in the DMN have dense white matter connections, suggesting that they form part of the brain’s structural core. Disruption of the DMN is clinically important, and abnormalities are observed in various neurological and psychiatric disorders [21]. Second, according to published data, the default mode is present as a baseline and is suspended during IED activity [28, 54]. Of note is that DMN was not detected in two patients (patients Nos. 1 and 5) and, when present, it showed modified activation, especially in the frontal lobe (patients Nos. 2, 3, 4, and 6). Patient No. 7, who has temporal focus epilepsy, showed a DMN with very clear frontal activation. A possible explanation is that the brain damage or long-term epileptic activity recorded inside the magnet does not allow activation (or at least in part) of the DMN.

With the advent of simultaneous EEG and fMRI coregistration, complex transient hemodynamic responses associated with ictal and interictal events can be mapped noninvasively. Nevertheless, in patients with epilepsy, EEG remains the mainstay method to investigate functional brain abnormalities and to reveal characteristic paroxystic events reflecting neuronal synchronization. A caveat to this is that a wrong model of individual geometry and conductivity of the brain and a limited number of EEG channels can influence estimation of the sources. HdEEG technologies were developed to enhance the poor spatial information content of EEG activity. In addition, constraining the source analysis of high spatial resolution EEG to a patient’s MRI provides more accurate localization of epileptic activity. The source localization on hdEEG data allows for precise mapping of IED activity when the same analysis on standard EEG fails. Furthermore, the sensitivity of inter-ictal or ictal EEG can be significantly increased with the use of additional zygomatic and pre-frontal electrodes.

The main objective of EEG-fMRI and hdEEG, as an independent electrophysiological and functional measurement, is to view brain function from various different perspectives and to detect and describe changes in neuronal activity. While IED recording and analysis of events can play a central role in the correct diagnosis of epilepsy, simple partial seizures may not have a definite location on scalp EEG. From EEG-fMRI and hdEEG registration, insights can be gained into networks associated with interictal epileptic activity [20]; moreover, having two different methods to localize the same epileptic foci is clinically useful. ESI and simultaneous EEG-fMRI are complementary methods for exploring epileptic activity. fMRI shows a complex network of focal hemodynamic changes related to interictal activity with high spatial resolution, whereas ESI identifies the source based on the electrical scalp activity and reveal the temporal time course of the regions involved during the spike event. The application of multiple methods to investigate the same events is an additional tool which can add sensitivity to the study. In particular, these post-traumatic epileptic patients showed several BOLD activations close to and far from the lesion. Part of these BOLD activations overlapped with the source activity in some cases but not in others, suggesting a more systemic activation or spread activations.

An area where EEG and fMRI integration and ESI have considerable clinical relevance is in the presurgical evaluation of epileptic patients: in many patients with drug-resistant focal seizures undergoing epilepsy surgery, standard magnetic resonance imaging scans fail to visualize a clear epileptic source, making preliminary invasive stereo EEG analysis necessary. Simultaneous EEG and fMRI recording and hdEEG offer a less invasive alternative, since they provide valuable information to localize the brain regions generating interictal epileptiform activity. In this group of TBI patients, who are not candidates for any surgical treatment, more information on the areas involved in the epileptic process are necessary in order to understand the physiopathological mechanisms.

On comparison of the hemodynamic and electrophysiological data, the results demonstrated a good correspondence in three out of seven patients (patients Nos. 1, 6, and 7), in three patients (patients Nos. 2, 3 and 5) no BOLD activation was found and in one patient (No. 4) the results were only partially related. The BOLD signal reflects the effect of electrical activity at the microvascular level, with high errors of measure (low signal-to-noise ratio), whereas imprecision in EEG source localization is related to an ill-posed nature of the problem, to errors of the head and source models. Other possible explanations may be sought in deep epileptic activities recognized by fMRI but not measured on scalp electrodes because of attenuation phenomena. Conversely, asynchronous neurons, which produce only minimal metabolic activities, can be measured by fMRI and not by EEG [45]. In other cases, the two signals can be negatively correlated. A high current density detected by the source localization is associated with hypo-hemodynamic activity in the same area.

Complementary information can be gleaned from a strategy that combines source analysis and coregistration, particularly in those forms of epilepsy in which clear focal activity is difficult to identify. A multimodal approach could be ideal for assessing patients with post-TBI epilepsy probably because of the long period of spiking alternating with rest periods and because patients with brain injury have sensitive metabolic changes in perilesional tissues. In such patients we cannot exclude changes in perfusion during interictal period due to the functional and anatomical modifications of vessels in a injured brain [35]. In addition the post-traumatic brain has phenomena of maladaptive plasticity due to fiber rearrangement after diffuse axonal injury that can be responsible for the epileptogenesis [53, 55].

## Conclusion

Noninvasive imaging techniques such as EEG-fMRI or hdEEG, separately considered, are widely used to investigate abnormal neural activity in relation to BOLD activity and electrical activity, respectively. EEG-fMRI can provide information on the pathophysiological processes underlying interictal activity, since the hemodynamic changes are a consequence of the abnormal neural activity generating IEDs; the source analysis estimates the current density of the source that generates a measured electric potential and it yields a plausible dipole localization of irritative regions. Furthermore, the combined use of EEG-fMRI and hdEEG offers a new and more complete approach to the study of epilepsy and may play an increasingly important role in the evaluation of patients with post-traumatic injury.
